# Three-Phase Decline in Reported Farm-Level Fowl Typhoid Occurrence in the Republic of Korea, 2000–2024: Temporal Alignment with the 2016–2017 H5N6 HPAI Epidemic

**DOI:** 10.3390/ani16172716

**Published:** 2026-09-01

**Authors:** Dae Sung Yoo, Yeonsu Oh

**Affiliations:** 1 Department of Veterinary Epidemiology, College of Veterinary Medicine and Animal Medical Institute, Chonnam National University, Gwangju 61186, Republic of Korea; shanuar@chonnam.ac.kr; 2 Department of Veterinary Pathology, College of Veterinary Medicine and Institute of Veterinary Science, Kangwon National University, Chuncheon 24341, Republic of Korea

**Keywords:** fowl typhoid, *Salmonella Gallinarum*, segmented regression, biosecurity, highly pathogenic avian influenza

## Abstract

This study characterized the long-term changes in reported farm-level fowl typhoid occurrence in the Republic of Korea from 2000 to 2024. Farm-level notification records (*n* = 1495) were obtained from the national livestock disease database. Annual counts were analyzed on a log(count + 1) scale using segmented linear regression. The number of breakpoints was selected by Bayesian information criterion (BIC) comparison of candidate segmented models, and BIC favored a two-breakpoint model. Breakpoints were estimated at 2009.0 (95% CI, 2004.7–2013.3) and 2016.5 (95% CI, 2014.1–2019.0). Phase 1 (2000–2009) declined significantly (annual percent change [APC], −16.3%; 95% CI, −25.9 to −5.5), Phase 2 (2009–2016) showed no significant change (APC, 3.7%; 95% CI, −10.3 to 20.0), and Phase 3 (2016–2024) showed an accelerated decline (APC, −28.0%; 95% CI, −34.9 to −19.8). Province-specific post-2016 slopes were not associated with cumulative provincial HPAI events during 2014–2024 (Spearman’s ρ = −0.143, *p* = 0.736). The post-2016 acceleration temporally overlapped with the 2016–2017 H5N6 HPAI epidemic, but the study did not measure the contemporaneous interventions or denominators needed for causal attribution.

## Simple Summary

Fowl typhoid is a serious bacterial disease of chickens that can cause high mortality and substantial economic losses. This study examined 1495 national farm-level notifications in the Republic of Korea from 2000 to 2024 to determine how the long-term reported trend changed over time. Formal model comparison using the Bayesian information criterion favored a pattern with two changes in slope, defining an initial decline from 2000 to 2009, a period of little overall change from 2009 to 2016, and a faster decline after 2016. The final phase overlapped in time with the large 2016–2017 H5N6 highly pathogenic avian influenza epidemic. However, provinces with more avian influenza outbreaks did not consistently show faster reductions in fowl typhoid. Because this study did not directly measure biosecurity intensity, vaccination coverage, surveillance effort, or changes in the poultry population and industry structure, it cannot identify the mechanism responsible for the post-2016 decline. The results therefore describe a long-term pattern in reported farm-level notifications and a temporal overlap with the HPAI epidemic period, rather than a causal effect of HPAI control measures.

## 1. Introduction

Fowl typhoid is a septicemic disease of chickens caused by *Salmonella enterica* subsp*. enterica* serovar *Gallinarum* biovar *Gallinarum* (*S. Gallinarum*), a host-adapted, nonmotile, Gram-negative bacterium [1,2]. The disease predominantly affects growing and adult chickens and is associated with high morbidity and mortality, reduced egg production, impaired fertility and hatchability, and substantial losses in commercial layer and breeder flocks [1]. Both vertical and horizontal transmission contribute to disease persistence, with transmission through infected eggs being particularly important for maintaining infection across generations [1,2]. A global analysis covering 1945–2021 estimated an overall prevalence of *S. Gallinarum* of 8.54%, with the highest burden reported in Asia [3].

In the Republic of Korea (ROK), fowl typhoid has been an important bacterial poultry disease since the early 1990s and remains within the national livestock-disease control framework. The live attenuated *S. Gallinarum* 9R (SG9R) vaccine was introduced for commercial layer chickens in 2001 as a preventive control option rather than as a universally compulsory vaccination program; its use has been sector-specific, and SG9R vaccination is not permitted in commercial broilers [4,5]. A previous Korean surveillance study reported that farm-level occurrence peaked at 206 affected farms in 2002 and subsequently declined in parallel with increased vaccine use [4]. Nevertheless, field strains of *S. Gallinarum* have continued to evolve in Korean poultry populations [6], and antimicrobial resistance among Korean isolates increased markedly between 2014 and 2018 [7]. Experimental studies have therefore evaluated safer SG9R derivatives and alternative live, adjuvanted, and inactivated vaccine candidates [8,9,10,11,12,13]. Annual vaccination coverage by the poultry sector was not available consistently for the full study period, preventing direct quantification of vaccination as a time-varying exposure in the present analysis.

Fowl typhoid and highly pathogenic avian influenza (HPAI) notifications are generated within Korea’s statutory animal-disease reporting and control system rather than through a single unchanging active-surveillance protocol. The Act on Prevention of Contagious Animal Diseases provides the legal framework for reporting suspected and confirmed livestock diseases and for official investigation and control [14]. In the public occurrence database, the recorded occurrence date corresponds to the diagnosis date and the diagnosing institution is identified, while national breeder-farm and hatchery guidelines provide an active component through scheduled pullorum disease/fowl typhoid testing of breeder flocks [15]. Under this official control program, breeder chickens confirmed positive for pullorum disease or fowl typhoid are subject to compulsory culling, and breeder flocks housed in affected poultry houses are subject to movement restrictions and restrictions on their continued use for breeding; eggs from such flocks are also restricted from use for hatching [15]. These measures are sector-specific and differ from the nationwide stamping-out approach applied to HPAI outbreaks. HPAI surveillance has included year-round active surveillance of poultry and wild birds in addition to passive reporting, and outbreak control has included diagnostic investigation, depopulation, movement restrictions, temporary standstill orders, and large-scale cleaning and disinfection [16,17]. During the 2016–2017 H5N6 epidemic, the ROK maintained a no-vaccination, stamping-out policy for HPAI [17]. Thus, surveillance and control intensity were not necessarily constant across the 25-year period or identical between diseases. The present study consequently treats the outcome as reported farm-level occurrence rather than incidence or infection risk. Routine quantitative data on surveillance effort, farm-level compliance, and fowl typhoid vaccination coverage were not available for adjustment.

Segmented, or joinpoint, regression provides a method for identifying points at which the slope of a temporal trend changes and for estimating phase-specific rates of increase or decline [18,19,20]. Unlike a simple comparison of arbitrarily defined periods, this approach estimates breakpoints from the observed data and expresses the resulting trends as annual percent changes (APCs), facilitating epidemiological and policy interpretation. Segmented regression and related interrupted time-series approaches have been widely used to evaluate population-level interventions and changes in disease or antimicrobial-resistance trends [21,22,23,24,25]. This approach is therefore well suited to long-term surveillance data in which multiple disease-control activities and major epidemic events may have altered transmission dynamics over time.

Measures introduced against one infectious agent can alter the transmission environment for other pathogens when routes or control practices overlap. Korean HPAI studies have documented farm-to-farm spread through vehicle movements and spatial risk associated with inland waters, poultry-farm distribution, wild-bird habitat, and broader ecological factors [26,27,28,29,30,31,32]. The 2016–2017 H5N6 HPAI epidemic was one of the largest poultry disease events recorded in the ROK, affecting 343 poultry farms and resulting in the culling of approximately 40 million birds [26]. Contemporaneous studies also documented H5N6 activity in Korean poultry workers and in birds in the wider regional epidemic context [33,34]. HPAI emergency responses can involve depopulation, movement restriction, cleaning and disinfection, and heightened surveillance, any of which could affect notifications of other poultry diseases directly or indirectly. Because the present dataset contains no direct measure of the intensity or compliance of these interventions, HPAI occurrence is used only as an epidemiological context and not as a quantitative proxy for biosecurity reinforcement.

Accordingly, this study aimed to characterize the national trajectory of reported farm-level fowl typhoid occurrence in the ROK from 2000 through 2024, formally select the number of temporal breakpoints, and estimate phase-specific annual percent changes (APCs). We also explored geographical heterogeneity in post-2016 provincial trends and assessed whether province-specific slopes were associated with cumulative HPAI occurrence. Particular attention was given to whether the timing of the final trend change overlapped with the 2016–2017 H5N6 HPAI epidemic. These analyses were intended to describe temporal and geographical patterns in notifications and to assess the compatibility of the timing with several plausible contemporaneous mechanisms, not to establish a causal effect of HPAI control.

## 2. Materials and Methods

### 2.1. Study Design and Data Sources

This retrospective ecological time-series study analyzed nationally reported farm-level fowl typhoid occurrence records in the ROK from 1 January 2000 to 31 December 2024. Fowl typhoid occurrence records were obtained from the livestock disease database of the Animal and Plant Quarantine Agency (APQA) through the public open application programming interface (API) provided by the Ministry of Agriculture, Food and Rural Affairs (MAFRA; data identifier: TI_QIA_LVSTCK_DISOCNC_INFO). Each database record represented a reported farm-level disease occurrence and included the event identifier, disease name, farm location, date of occurrence, livestock species and breed, number of affected animals, diagnostic institution, and date of case closure.

A total of 1495 farm-level fowl typhoid records were identified during the study period. For the exploratory province-level analysis, farm-level HPAI occurrence records from 2014 to 2024 were retrieved from the same APQA livestock disease database. HPAI records were aggregated by province to calculate the cumulative number of reported farm-level events during this period.

### 2.2. Data Retrieval and Processing

Data were retrieved in May 2026 through paginated API requests, with a maximum of 1000 records obtained per request, using the httr package version 1.4.8 in R [35]. Two Korean orthographic variants of the disease name were identified in the fowl typhoid records. Both variants referred to the same notifiable disease and were harmonized into a single fowl typhoid category using regular-expression matching implemented in the stringr package version 1.6.0 [36].

Farm-level records were aggregated by calendar year to obtain the annual number of reported fowl typhoid occurrences. All calendar years from 2000 to 2024 were included, and years without reported occurrences were assigned a count of zero. Province-level annual counts were generated using the first-level administrative unit recorded for each farm.

For descriptive visualization, annual province-specific counts were displayed as a heatmap using a log(count + 1) scale. Provinces were ordered according to their total number of reported fowl typhoid occurrences during the full study period. Province-level analyses were restricted to provinces with at least 20 reported occurrences during 2000–2024 to reduce instability arising from sparse annual series.

### 2.3. Segmented Regression Analysis of the National Trend

The primary outcome was the annual number of reported farm-level fowl typhoid occurrences. Annual occurrence counts were transformed as log(count + 1) to stabilize variance and accommodate years with low or zero counts.

The number of breakpoints was not fixed a priori. Candidate models with zero, one, and two breakpoints were compared using the Bayesian information criterion (BIC), and the two-breakpoint model had the lowest BIC. This model-selection step preceded interpretation of the three temporal phases. The initial values subsequently supplied for numerical estimation were starting values only and were not used to determine the number of breakpoints.

Breakpoint locations and phase-specific slopes in the BIC-selected model were estimated using the segmented R package version 2.2-1 [19,20]. Initial values of 2005 and 2015 were supplied only as starting values for numerical optimization and did not determine model selection or constrain the final breakpoint locations. Approximate 95% confidence intervals (CIs) for breakpoint locations were obtained using the CI procedure implemented in the package.

The selected two-breakpoint segmented model was expressed as follows:

log(count_t_ + 1) = β_0_ + β_1_t + ∑_k_ U_k_ max(0, t − ψ_k_) + ε_t_

where count_t_ denotes the annual number of reported farm-level occurrences in year t, ψ_k_ denotes the location of the kth breakpoint, U_k_ denotes the corresponding change-in-slope coefficient at ψ_k_, and ε_t_ denotes the residual error.

The APC for each phase was calculated from its estimated log-scale slope as follows:

$$APC=\left[exp\left(\beta m\right)-1\right]\times 100$$

where βm denotes the phase-specific slope. The 95% CIs for the APC estimates were obtained by applying the same exponential transformation to the lower and upper confidence limits of the corresponding slopes. Phase-specific slopes were tested against zero using model-based standard errors.

**Figure 1:**
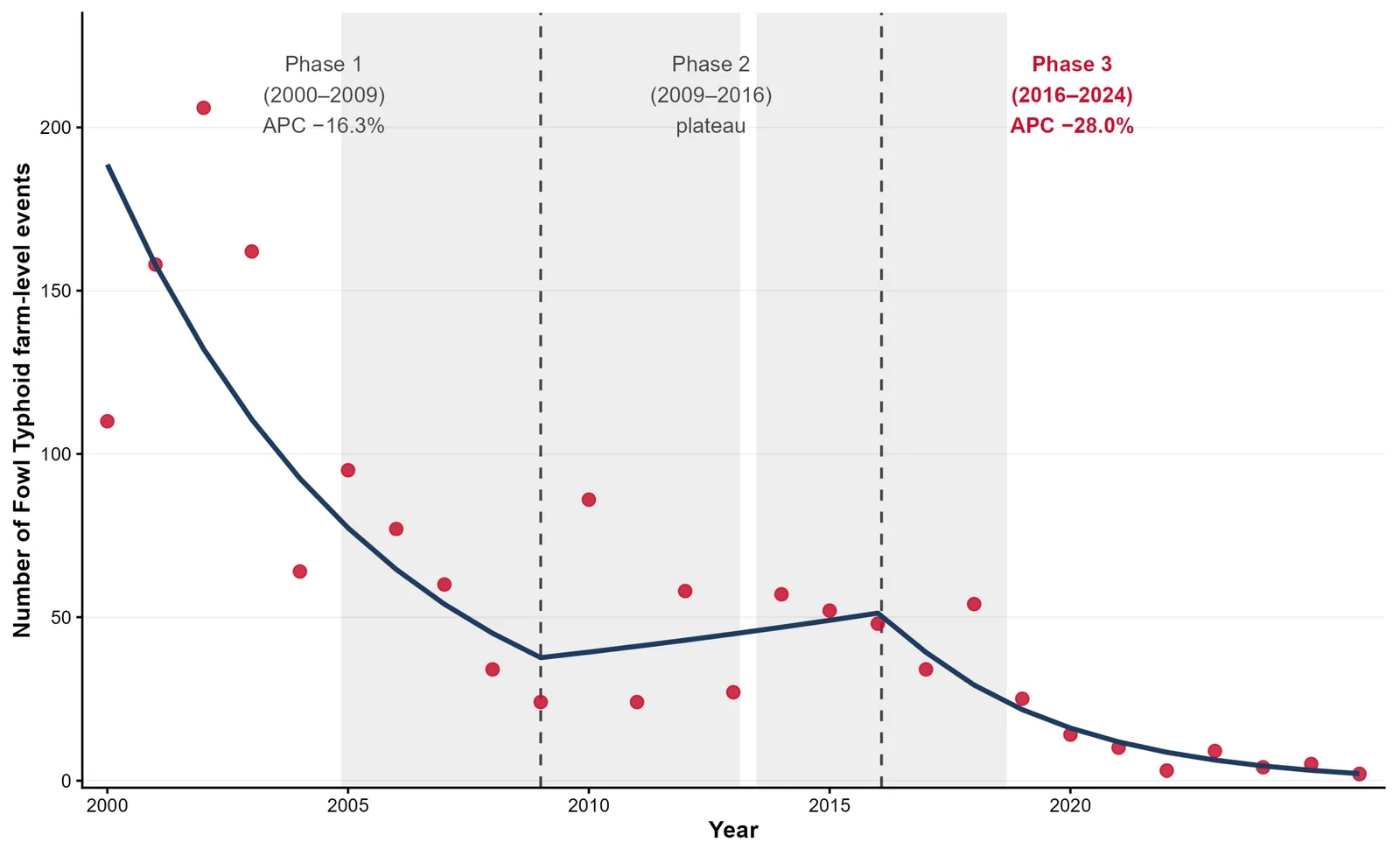
BIC-selected three-phase national trend in reported farm-level fowl typhoid occurrence in the Republic of Korea, 2000–2024. Red points represent the annual numbers of reported farm-level occurrences. The solid blue line shows fitted values from the segmented linear regression model of log(count + 1), displayed on the original count scale as exp(ŷ) − 1 for visualization. Vertical dashed lines indicate the estimated breakpoints at 2009.0 and 2016.5. Shaded vertical bands indicate approximate 95% confidence intervals for breakpoint locations and represent uncertainty in breakpoint timing; they do not denote intervention periods. APC estimates are shown for the declining phases; the Phase 2 slope was not statistically significant.

The Davies test was used as a complementary test for evidence of at least one change in slope [24]; it was not used to select the number of breakpoints. Model adequacy was evaluated using the adjusted coefficient of determination (R^2^), residual standard error, and visual inspection of residual diagnostic plots. For Figure 1, fitted log-scale values were transformed as exp(ŷ) − 1 solely to display the fitted trajectory on the original count scale. This transformation was used for visualization and was not interpreted as an unbiased prediction of the arithmetic mean count; all slope estimates, hypothesis tests, and APC inference were conducted on the log(count + 1) scale. The temporal alignment between estimated breakpoints and documented Korean poultry health events was examined descriptively.

### 2.4. Exploratory Province-Level Analysis

To examine geographical heterogeneity in the post-2016 trajectory, separate linear regression models were fitted for each province using annual fowl typhoid counts from 2016 to 2024. The dependent variable was log(count + 1), and calendar year was entered as the independent variable. The estimated regression coefficient for calendar year was used as the province-specific post-2016 slope. Negative slopes indicated declining occurrence, with increasingly negative values representing more rapid reductions, whereas positive slopes indicated stable or increasing occurrence.

The analysis included eight provinces with at least 20 reported fowl typhoid occurrences during 2000–2024. To describe geographical consistency with the HPAI epidemic context, province-specific post-2016 slopes were compared with the cumulative number of reported farm-level HPAI events in each province during 2014–2024. Cumulative HPAI event count was treated only as an ecological contextual variable and not as a measure of biosecurity intensity, policy implementation, or compliance.

Spearman’s rank correlation was used because of the small number of provinces and because a linear relationship between HPAI occurrence and the rate of decline in reported fowl typhoid occurrence could not be assumed. In the corresponding scatter plot, an ordinary least-squares regression line and its 95% CI were included solely to visualize the direction and uncertainty of the association; statistical inference was based on Spearman’s correlation. Because only eight provinces were included, this analysis was considered exploratory and was not interpreted as a causal evaluation of the effects of HPAI control measures or other contemporaneous interventions.

### 2.5. Statistical Software

All data processing, statistical analyses, and graphical summaries were conducted using R software version 4.4.0 [37]. All statistical tests were two-sided, and values of *p* < 0.05 were considered statistically significant. Generative AI was not used for data processing or statistical analysis. 

## 3. Results

### 3.1. Descriptive Distribution of Fowl Typhoid Occurrence

A total of 1495 reported farm-level fowl typhoid occurrence records were identified in the Republic of Korea during 2000–2024. Annual reported occurrences were relatively high in the early 2000s, followed by an overall long-term reduction. However, the decline was not uniform throughout the study period, with an interval of limited change during the middle years and a more pronounced reduction after 2016 (Figure 1).

**Figure 2:**
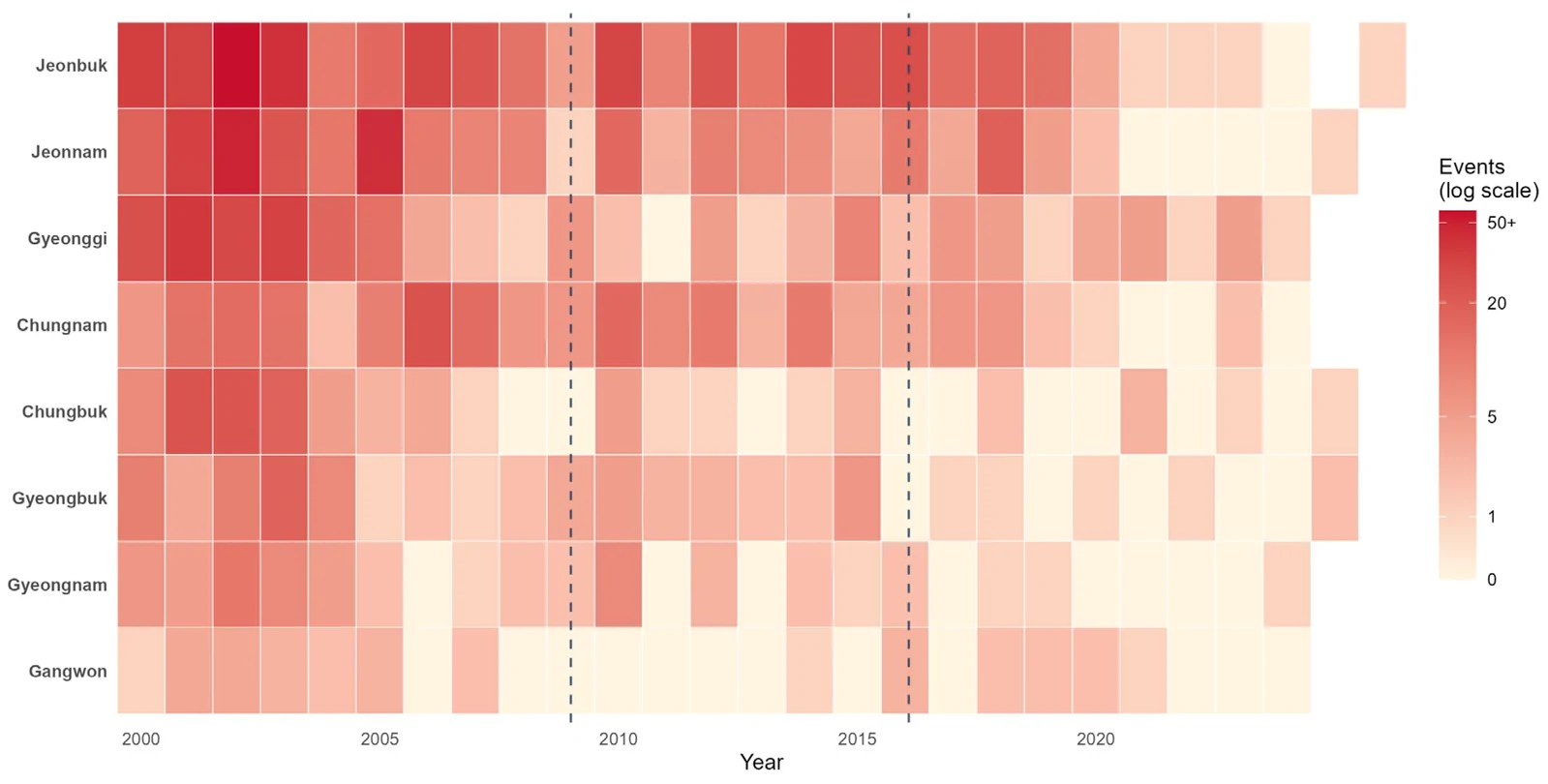
Year-by-province distribution of reported farm-level fowl typhoid occurrences in the Republic of Korea, 2000–2024. Cell shading represents annual province-specific occurrence counts transformed as log(count + 1), with darker shading indicating higher reported occurrences. Provinces are ordered according to their total number of reported occurrences during the study period. Only provinces with at least 20 reported occurrences during 2000–2024 are displayed. Vertical dashed lines indicate the estimated national breakpoints at 2009.0 and 2016.5. The sequential light-to-dark color scale was selected so that magnitude is conveyed primarily by luminance.

Reported occurrences were geographically concentrated in the major poultry-producing provinces, particularly Jeollabuk-do, Jeollanam-do, and Chungcheongnam-do. The year-by-province heatmap showed substantial geographical heterogeneity in both the magnitude and temporal pattern of occurrence. Most provinces exhibited a reduction in reported occurrences over time, although the timing and extent of the decline differed among provinces (Figure 2).

### 3.2. Breakpoints and Phase-Specific National Trends

BIC-based model selection comparing zero-, one-, and two-breakpoint candidate models favored the two-breakpoint specification, which had the lowest BIC. Within the selected model, the first breakpoint was estimated at 2009.0 (95% confidence interval [CI], 2004.7–2013.3), and the second at 2016.5 (95% CI, 2014.1–2019.0). These estimated breakpoints defined three phases (Figure 1). The Davies test provided complementary evidence for at least one change in the slope of the national trend (*p* < 0.001) but was not used to select the number of breakpoints.

Phase 1, covering 2000–2009, showed a significant decline, with an APC of −16.3% (95% CI, −25.9 to −5.5; *p* = 0.006). Phase 2, covering 2009–2016, showed no significant temporal change, with an APC of 3.7% (95% CI, −10.3 to 20.0; *p* = 0.604). Phase 3, covering 2016–2024, showed the most rapid reduction, with an APC of −28.0% (95% CI, −34.9 to −19.8; *p* < 0.001). The absolute magnitude of the Phase 3 APC was approximately 1.7 times that observed during Phase 1 (Table 1).

### 3.3. Model Adequacy

The segmented regression model explained 87% of the variation in annual log-transformed occurrence counts, with an adjusted $R^{2}$ of 0.87. The residual standard error was 0.45 on 21 degrees of freedom. Visual inspection of the residual diagnostic plots showed no evident systematic departures from the model assumptions.

### 3.4. Province-Level Post-2016 Trends and Association with HPAI Occurrence

Province-specific slopes for 2016–2024 demonstrated geographical heterogeneity in post-2016 fowl typhoid trends. Six of the eight included provinces showed negative slopes, indicating declining occurrence. The steepest reductions were observed in Jeollabuk-do and Jeollanam-do, whereas Chungcheongbuk-do and Gyeongsangbuk-do showed slightly positive slopes (Figure S1).

The cumulative number of reported farm-level HPAI events in each province during 2014–2024 was not significantly associated with the province-specific post-2016 fowl typhoid slope (Spearman’s ρ = −0.143, *p* = 0.736; Figure S2). Thus, provinces with a greater cumulative number of HPAI events did not consistently exhibit faster reductions in reported fowl typhoid occurrence.

## 4. Discussion

This study showed that reported farm-level fowl typhoid occurrence in the Republic of Korea followed a BIC-supported three-phase temporal pattern. A significant decline occurred during 2000–2009, followed by a period with no significant trend during 2009–2016 and a substantially faster decline during 2016–2024. The annual rate of reduction in the final phase was approximately 1.7 times greater in absolute magnitude than that observed during the initial declining phase. The estimated second breakpoint overlapped temporally with the 2016–2017 H5N6 HPAI epidemic, but its 95% CI extended from 2014.1 to 2019.0. Moreover, cumulative provincial HPAI occurrence was not associated with province-specific post-2016 fowl typhoid slopes. The findings therefore establish a change in the temporal pattern of reported notifications, but they do not identify the mechanism responsible for that change.

The Phase 1 decline was temporally consistent with the introduction and expansion of the national fowl typhoid vaccination program. The live attenuated *S. Gallinarum* 9R vaccine was introduced for commercial layer chickens in 2001, and a previous Korean study reported that farm-level occurrence peaked in 2002 and subsequently declined in parallel with increased vaccine use [4]. Vaccination, surveillance, breeder-flock management, and improvements in farm hygiene may all have contributed to the early reduction. However, annual vaccination coverage by sector was unavailable across the full study period, and the present ecological analysis cannot quantify the independent contribution of vaccination or determine whether changes in vaccine coverage contributed to the intermediate plateau or post-2016 decline.

The absence of a significant downward trend during Phase 2 suggests that the initial reduction was followed by a period of limited further progress. Although the estimated annual percent change was slightly positive, its confidence interval was wide and included zero; therefore, this phase should be interpreted as a plateau rather than a true resurgence. Persistent low-level transmission may have resulted from vertical transmission, infected breeder populations, uneven vaccination coverage, small-scale poultry holdings, or incomplete implementation of biosecurity measures [1,2]. Korean molecular studies also showed continued diversification of field *S. Gallinarum* strains [6], and antimicrobial resistance increased among isolates collected between 2014 and 2018 [7]. These findings indicate that reduced national occurrence did not necessarily represent complete interruption of transmission.

The accelerated decline during Phase 3 was the most prominent change in the fitted trend. The second breakpoint was estimated at 2016.5, but the broad 95% CI (2014.1–2019.0) means that it should not be interpreted as a precise intervention date. Its timing nevertheless overlapped with the 2016–2017 H5N6 HPAI epidemic and a period of intensive HPAI response. Such responses included depopulation of affected premises, movement controls, cleaning and disinfection, and heightened surveillance [16,26,27]. *S. Gallinarum* and HPAI viruses can both be disseminated between farms through contaminated footwear, equipment, vehicles, personnel, and other mechanically contaminated materials [2,27,38,39,40,41]. Accordingly, some HPAI-related control activities are biologically compatible with reduced transmission opportunities for fowl typhoid, but this is only one of several plausible explanations and was not directly measured in this study.

Large-scale depopulation provides an important alternative or complementary mechanism. Removal of affected poultry populations could temporarily reduce the population at risk, eliminate premises harboring recognized or unrecognized concurrent *S. Gallinarum* infection, generate periods of sanitary downtime, and contribute to subsequent farm closure, consolidation, or changes in production structure. Movement restrictions on poultry-related vehicles, personnel, and facilities can also alter contact networks; importantly, temporary standstill and mass-disinfection measures were already used in Korea before 2016 [16], indicating that poultry biosecurity policy evolved over time rather than beginning at a single breakpoint. Changes in vaccination practice, flock replacement, farm density, surveillance intensity, diagnostic submissions, and resource allocation during HPAI emergencies could likewise increase or decrease the number of fowl typhoid notifications. Because consistent longitudinal measures of these factors were unavailable, their contributions cannot be separated in the present analysis.

The province-level analysis further argues against treating HPAI outbreak burden as a simple proxy for the mechanism of the fowl typhoid decline. Six of the eight included provinces showed declining post-2016 trends, but provinces with more HPAI events did not consistently show faster reductions in reported fowl typhoid occurrence. HPAI event counts do not measure the intensity, duration, geographical reach, or compliance of subsequent control measures, and nationwide policies can affect farms outside locally affected provinces. Regional poultry density, production type, vaccination practices, surveillance intensity, baseline fowl typhoid burden, depopulation, and industry restructuring could also influence provincial trends [28,29,30,31,32]. With only eight provinces, the correlation analysis had limited statistical power and remains exploratory.

Indirect effects of broad transmission-control measures on non-target infectious diseases have been documented in other settings. During the COVID-19 pandemic, a controlled interrupted time-series study using national surveillance data in China found substantial reductions in several notifiable infectious diseases after non-pharmaceutical interventions were introduced [42], and a nationwide French analysis reported a marked decrease in pertussis during COVID-19 mitigation measures [43]. These studies demonstrate the general epidemiological plausibility that interventions targeting one pathogen can affect other infections when relevant transmission opportunities are simultaneously reduced. They do not, however, provide evidence that HPAI control caused the post-2016 fowl typhoid decline in Korea. Testing that hypothesis would require direct measures of biosecurity implementation, depopulation intensity, vaccination coverage, farm population denominators, movement networks, and surveillance effort.

Several limitations are central to interpretation. First, the APQA database contains reported farm-level notifications rather than incidence estimates from a stable active surveillance denominator. Changes in diagnostic activity, reporting behavior, surveillance effort, or allocation of field resources could therefore change notification counts independently of infection risk. Second, consistent annual denominators for the number of operating poultry farms, flocks, birds, flock turnover, farm size, and population-sector composition were unavailable. The outcome should consequently be interpreted as the number of reported farm-level occurrences, not incidence or risk. Third, longitudinal data on vaccination coverage, farm-level biosecurity implementation and compliance, depopulation intensity, movement restrictions, and industry restructuring were not available for multivariable adjustment. Fourth, cumulative HPAI event count is an ecological contextual measure and not a quantitative exposure to HPAI control. Fifth, although the public database contains farm names and administrative locations, it does not provide a persistent longitudinal premises identifier suitable for reliable cross-disease linkage across years; therefore, a farm-level HPAI-fowl typhoid overlap analysis was not undertaken because name/address linkage could introduce substantial misclassification. Finally, the national time series is relatively short, and the breakpoint confidence intervals—particularly for the second breakpoint—indicate appreciable uncertainty in the exact timing of slope changes.

## 5. Conclusions

Reported farm-level fowl typhoid notifications in the ROK followed a BIC-supported three-phase pattern from 2000 to 2024, consisting of an initial decline, an intermediate plateau, and an accelerated decline after 2016. The estimated timing of the final trend change overlapped with the 2016–2017 H5N6 HPAI epidemic, but the study did not directly measure biosecurity implementation or other contemporaneous changes. Depopulation, movement restrictions and sanitation, vaccination practices, surveillance changes, and poultry-industry restructuring are all plausible contributors that could not be disentangled with the available ecological data. Province-level HPAI occurrence was not associated with post-2016 fowl typhoid slopes. The findings therefore establish a temporal pattern in reported notifications, not a causal effect of HPAI control. Future analyses incorporating stable denominators, direct intervention-exposure measures, and reliable premises-level linkage are needed to clarify the mechanisms underlying the long-term decline.

## Figures and Tables

**Table 1:** Phase-specific slope estimates, annual percent change (APC), and 95% confidence intervals from segmented linear regression of log-transformed annual fowl typhoid reported occurrence counts in the Republic of Korea, 2000–2024.

Phase	Period	Slope β (95% CI)	APC, % (95% CI)	*p* Value
1	2000–2009	−0.178 (−0.300 to −0.057)	−16.3 (−25.9 to −5.5)	0.006
2	2009–2016	0.036 (−0.109 to 0.182)	3.7 (−10.3 to 20.0)	0.604
3	2016–2024	−0.328 (−0.432 to −0.225)	−28.0 (−34.9 to −19.8)	<0.001

Note: CI, confidence interval. Slope β denotes the phase-specific coefficient from segmented linear regression of log(count + 1) on calendar year; APC = [exp(β) − 1] × 100. BIC-based comparison of candidate models selected the two-breakpoint specification. The estimated breakpoints were 2009.0 (95% CI, 2004.7–2013.3) and 2016.5 (95% CI, 2014.1–2019.0). Overall model fit: adjusted R^2^ = 0.87. Estimates were obtained using the segmented R package, version 2.2-1.

## Data Availability

The farm-level occurrence records analyzed in this study are publicly available through the Ministry of Agriculture, Food and Rural Affairs public data portal under dataset identifier TI_QIA_LVSTCK_DISOCNC_INFO. The processed annual dataset and analysis code are available from the corresponding author upon reasonable request.

## Supplementary Materials

The following supporting information can be downloaded at: https://www.mdpi.com/article/10.3390/ani16172716/s1, Table S1: Annual reported farm-level fowl typhoid occurrence counts in the Republic of Korea, 2000–2024; Table S2: Annual province-specific reported farm-level fowl typhoid occurrence counts underlying Figure 2, 2000–2024; Figure S1: Province-specific post-2016 trends in reported fowl typhoid occurrence; Figure S2: Association between cumulative provincial highly pathogenic avian influenza events and post-2016 fowl typhoid trends.
