# Supplementary material for: Three-Phase Decline in Reported Farm-Level Fowl Typhoid Occurrence in the Republic of Korea, 2000–2024: Temporal Alignment with the 2016–2017 H5N6 HPAI Epidemic
Source: Animals (Basel). 2026 Sep 1;16(17):2716. doi: 10.3390/ani16172716 (PMC13565467; doi:10.3390/ani16172716)
Supplement: Supplementary file 1 [file animals-16-02716-s001.zip › animals-4467201-supplementary.pdf]

animals

Supplementary Materials

Three-Phase Decline in Reported Farm-Level Fowl Typhoid Occurrence in the Republic of Korea, 2000–2024: Temporal Alignment with the 2016–2017 H5N6 HPAI Epidemic

Dae-Sung Yoo and Yeonsu Oh *

* Correspondence: yeonoh@kangwon.ac.kr

Table S1. Annual reported farm-level fowl typhoid occurrence counts in the Republic of Korea, 2000–2024. (National annual fowl typhoid counts)

Year	Reported farm-level occurrences
2000	110
2001	158
2002	206
2003	162
2004	64
2005	95
2006	77
2007	60
2008	34
2009	24
2010	86
2011	24
2012	58
2013	27
2014	57
2015	52
2016	48
2017	34
2018	54
2019	25
2020	14
2021	10
2022	3
2023	9
2024	4

Table S2. Annual province-specific reported fowl typhoid occurrence counts underlying Figure 2, 2000–2024.

Year / Province	2000	2001	2002	2003	2004	2005	2006	2007	2008	2009	2010	2011	2012	2013	2014	2015	2016	2017	2018	2019	2020	2021	2022	2023	2024
Gyeonggi	26	38	29	33	17	14	4	2	1	6	2	0	5	1	3	9	2	6	5	1	4	5	1	5	1
Gangwon	1	4	4	3	2	3	0	2	0	0	0	0	0	0	1	0	3	0	2	2	2	1	0	0	0
Chungnam	6	13	15	13	2	10	25	15	6	6	16	8	11	3	11	4	4	6	6	2	1	0	0	2	0
Chungbuk	8	24	23	18	5	3	4	1	0	0	5	1	1	0	1	3	0	0	2	0	0	3	0	1	0
Jeonbuk	34	32	57	42	11	16	31	23	13	5	31	9	24	12	30	24	26	15	18	14	4	1	1	1	0
Jeonnam	18	33	49	23	12	43	11	9	9	1	16	3	10	8	7	4	11	4	19	5	2	0	0	0	0
Gyeongbuk	10	4	10	18	8	1	2	1	2	4	5	3	3	2	2	6	0	1	1	0	1	0	1	0	0
Gyeongnam	6	5	12	8	5	2	0	1	2	2	8	0	3	0	2	1	2	0	1	1	0	0	0	0	1

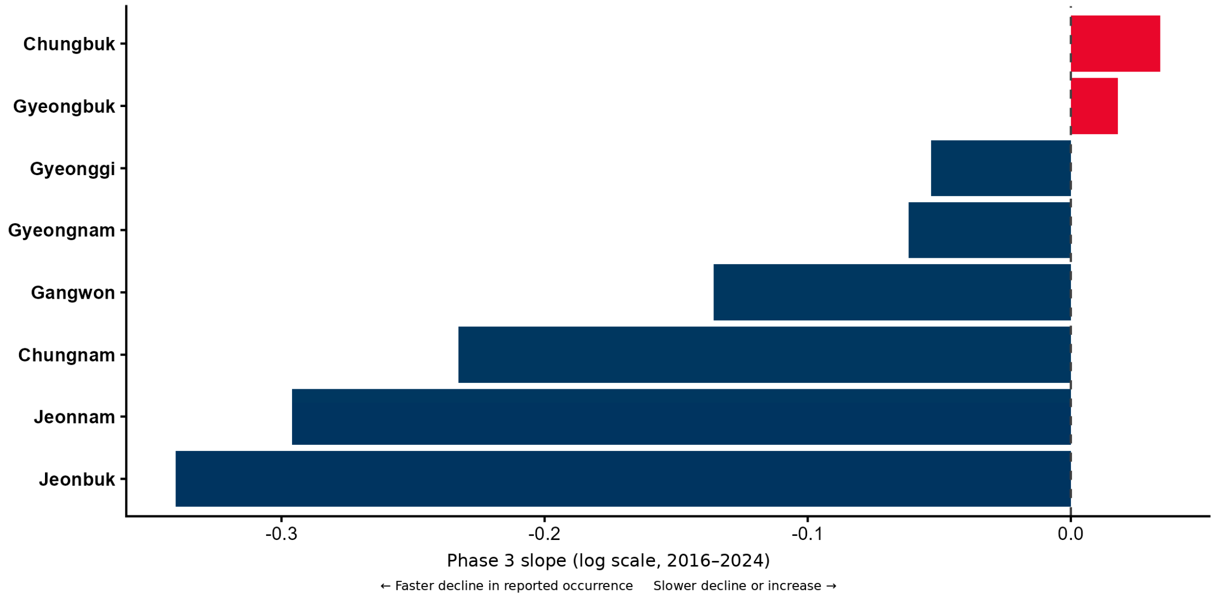

Figure S1. Province-specific post-2016 trends in reported fowl typhoid occurrence. Bars represent slopes estimated from separate linear regression models of log(count + 1) on calendar year during 2016–2024 for the eight included provinces. Negative slopes indicate declining reported occurrence, with more negative values representing faster declines, whereas positive slopes indicate stable or increasing reported occurrence. Provinces with at least 20 reported fowl typhoid occurrences during 2000–2024 were included.

https://doi.org/10.3390/ani16172716

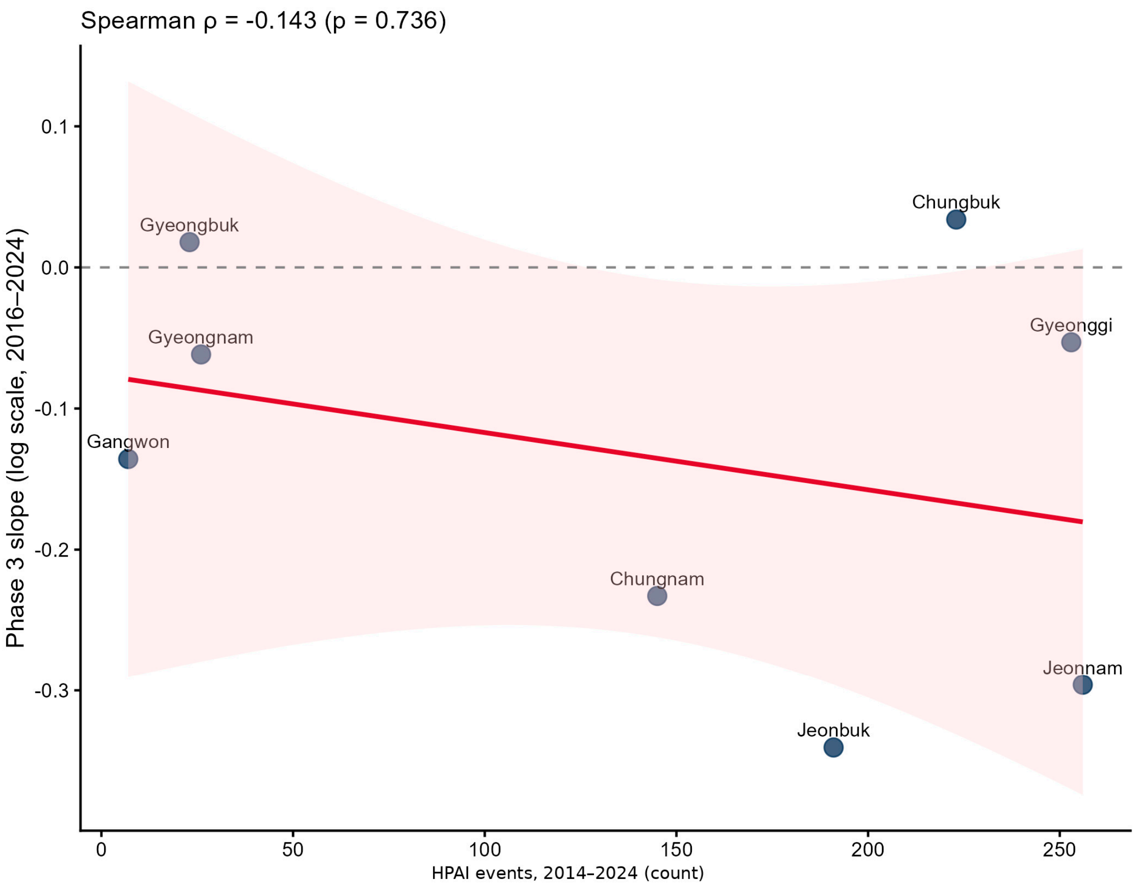

Figure S2. Association between cumulative provincial highly pathogenic avian influenza events and post-2016 fowl typhoid trends. Each point represents one province. The x-axis indicates the cumulative number of reported farm-level highly pathogenic avian influenza events during 2014–2024, and the y-axis indicates the province-specific slope estimated from linear regression of log(count + 1) on calendar year during 2016–2024. More negative slopes indicate faster declines in reported fowl typhoid occurrence. The solid line and shaded area represent the ordinary least-squares regression line and its 95% confidence interval, respectively, and are shown for visualization only. The horizontal dashed line indicates a slope of zero. Statistical inference was based on Spearman's rank correlation ($\varrho = -0.143$, $p = 0.736$).
